# Characterizing bleeding adverse events associated with BCR-ABL tyrosine kinase inhibitors: insights from FAERS reports

**DOI:** 10.3389/fonc.2026.1912458

**Published:** 2026-09-09

**Authors:** Ranran Liang, Lin Zhang, Yanlin Liu, Houli Zhang, Zhenshan Li, Chunxiao Wang

**Affiliations:** 1 Department of Pharmacy, The Second Afffliated Hospital of Shandong First Medical University, Taian, Shandong, China; 2 Medical Affairs Department, The Second Afffliated Hospital of Shandong First Medical University, Taian, Shandong, China

**Keywords:** BCR-ABL tyrosine kinase inhibitors, bleeding, chronic myeloid leukemia, FAERS, hemorrhage, pharmacovigilance

## Abstract

**Background:**

Bleeding is a potentially serious but underrecognized adverse event associated with BCR-ABL tyrosine kinase inhibitors (TKIs). Although hematologic and cardiovascular toxicities of these agents have been extensively investigated, the spectrum and characteristics of bleeding-related adverse events in real-world settings remain incompletely understood. This study aimed to characterize bleeding-related adverse event signals associated with five BCR-ABL TKIs using the U.S. Food and Drug Administration Adverse Event Reporting System (FAERS).

**Methods:**

A pharmacovigilance study was conducted using FAERS data from 2004Q1 to 2026Q1. Reports involving imatinib, dasatinib, nilotinib, ponatinib, and bosutinib as primary suspected drugs were identified. Bleeding-related adverse events were screened using Medical Dictionary for Regulatory Activities (MedDRA) Preferred Terms (PTs). Disproportionality analysis was performed using the reporting odds ratio (ROR) method to detect potential bleeding signals. Demographic characteristics, clinical outcomes, and distributions of bleeding-related signals across System Organ Classes (SOCs) were further evaluated.

**Results:**

Among 20, 326, 782 deduplicated FAERS reports, 124, 797 reports involving BCR-ABL TKIs were identified, including 3, 648 bleeding cases and 121, 149 non-bleeding cases. Bleeding reports were associated with higher proportions of hospitalization (19.8% vs. 14.4%) and life-threatening outcomes (2.7% vs. 1.5%) than non-bleeding reports. A total of 64 positive bleeding-related signals were detected across the five study drugs. Gastrointestinal disorders, nervous system disorders, and eye disorders represented the most frequently involved organ systems. Gastrointestinal haemorrhage, cerebral haemorrhage, and eye haemorrhage were recurrent signals detected across multiple TKIs. Several gastrointestinal bleeding-related signals were identified for dasatinib, including enterocolitis haemorrhagic (ROR = 28.98), while multiple bleeding-related PTs involving diverse organ systems were detected for imatinib. In addition, several uncommon PTs demonstrated elevated disproportionality estimates, although these findings were based on relatively small numbers of reports.

**Conclusions:**

Bleeding-related adverse events associated with BCR-ABL TKIs involve multiple organ systems and encompass a broad spectrum of clinical manifestations. Gastrointestinal, neurological, and ocular haemorrhagic events accounted for the majority of detected signals. These findings provide real-world evidence regarding the characteristics of bleeding-related adverse events associated with BCR-ABL TKIs and may contribute to pharmacovigilance monitoring, individualized safety management, and future investigations of haemorrhagic toxicity during TKI therapy.

## 1 Introduction

The advent of BCR-ABL tyrosine kinase inhibitors (TKIs) has revolutionized the treatment landscape of chronic myeloid leukemia (CML) and Philadelphia chromosome-positive acute lymphoblastic leukemia (Ph+ALL). Since the introduction of imatinib, the first-generation BCR-ABL TKI, CML has been transformed from a fatal hematologic malignancy into a chronic and manageable disease, resulting in substantial improvements in long-term survival and quality of life (1, 2). To overcome resistance and intolerance associated with imatinib, second-generation TKIs, including dasatinib, nilotinib, and bosutinib, as well as the third-generation TKI ponatinib, have been developed and widely adopted in clinical practice (3–6). These agents have become the cornerstone of personalized therapy for patients with CML and Ph+ALL.

Despite their remarkable therapeutic efficacy, BCR-ABL TKIs are associated with a broad spectrum of adverse events that may compromise treatment adherence and clinical outcomes (7). Among these toxicities, bleeding complications represent a clinically important but often underrecognized safety concern. Previous studies have reported bleeding events ranging from mild mucocutaneous hemorrhage to severe gastrointestinal and intracranial bleeding in patients receiving BCR-ABL TKIs, particularly dasatinib (8). The underlying mechanisms are multifactorial and may involve thrombocytopenia, platelet dysfunction, endothelial injury, and coagulation abnormalities (8, 9). In some cases, bleeding events may lead to hospitalization, treatment interruption, dose reduction, or even death, highlighting the need for enhanced pharmacovigilance and risk assessment.

Although bleeding events associated with individual BCR-ABL TKIs have been described in clinical trials, observational studies, and case reports, comprehensive evaluations of bleeding-related adverse event profiles across different BCR-ABL TKIs in real-world clinical practice remain limited. Furthermore, the spectrum of bleeding manifestations involving different organ systems has not been systematically characterized using large-scale pharmacovigilance data. The U.S. Food and Drug Administration Adverse Event Reporting System (FAERS), one of the largest spontaneous reporting databases worldwide, provides a valuable resource for detecting rare, serious, and unexpected adverse drug reactions that may not be fully captured in pre-marketing clinical trials (10).

Therefore, the present study aimed to comprehensively characterize bleeding-related adverse events associated with five BCR-ABL TKIs (imatinib, dasatinib, nilotinib, bosutinib, and ponatinib) using the FAERS database. We evaluated demographic characteristics, clinical outcomes, and bleeding signal profiles across different organ systems to provide real-world evidence supporting bleeding risk assessment, pharmacovigilance monitoring, and individualized safety management during BCR-ABL TKI therapy.

## 2 Materials and methods

### 2.1 Data source and study design

This pharmacovigilance study was conducted using data obtained from the U.S. Food and Drug Administration (FDA) Adverse Event Reporting System (FAERS), covering the period from the first quarter of 2004 (2004Q1) to the first quarter of 2026 (2026Q1). FAERS is a publicly accessible spontaneous reporting database designed to support post-marketing drug safety surveillance and contains information on patient demographics, drug exposure, adverse events, treatment duration, and clinical outcomes (11).

The quarterly FAERS datasets were downloaded from the FDA website and integrated for analysis. Data management and statistical analyses were performed using R software (version 4.2.3; R Foundation for Statistical Computing, Vienna, Austria). The DEMO, DRUG, REAC, THER, and OUTC tables were linked using the primary identification number (PRIMARYID) and case identification number (CASEID).

To improve data quality and avoid duplicate counting, reports were deduplicated according to the FDA-recommended procedure. When multiple records shared the same CASEID, the report with the most recent FDA receipt date (FDA_DT) was retained. If multiple reports had the same CASEID and FDA_DT, the record with the highest PRIMARYID was selected (12). After deduplication, the resulting dataset was used for subsequent analyses.

All adverse events were coded according to the Medical Dictionary for Regulatory Activities (MedDRA, version 28.1), and adverse event terms were standardized using the corresponding Preferred Terms (PTs) and System Organ Classes (SOCs).

### 2.2 Identification of BCR-ABL TKI reports

Reports involving BCR-ABL tyrosine kinase inhibitors (TKIs) were identified using both generic and brand names, including imatinib (Gleevec), dasatinib (Sprycel), nilotinib (Tasigna), bosutinib (Bosulif), and ponatinib (Iclusig). To improve the specificity of drug–event associations, only reports in which the target TKI was designated as the primary suspected (PS) drug were included in the analysis. Reports in which these agents were recorded as secondary suspected (SS), concomitant (C), or interacting (I) drugs were excluded.

### 2.3 Identification of bleeding events

Adverse events recorded in FAERS are coded according to the Medical Dictionary for Regulatory Activities (MedDRA, version 28.1). All adverse events were standardized and mapped to the corresponding Preferred Terms (PTs) and System Organ Classes (SOCs).

Bleeding-related adverse events were identified using a predefined PT screening strategy. Initially, MedDRA PTs containing bleeding-related keywords, including “haemorrhage”, “hemorrhage”, “bleeding”, “haematoma”, “hematoma”, “haematemesis”, “haematochezia”, “melaena”, “epistaxis”, and other clinically relevant haemorrhagic terms, were retrieved. Subsequently, all candidate PTs were independently reviewed by two investigators to exclude non-relevant events and ensure clinical relevance. Discrepancies regarding PT inclusion were resolved through discussion until consensus was reached.

The final list of bleeding-related PTs is provided in **Supplementary Table 1**. To characterize the anatomical distribution of bleeding manifestations, included PTs were further classified according to their corresponding SOCs. Signal detection analyses were subsequently performed at both the PT and SOC levels.

### 2.4 Data collection and statistical analysis

For each eligible report, demographic and clinical characteristics were extracted from the FAERS database, including sex, age, body weight, reporter type, reporting year, reporting country, and clinical outcomes. Descriptive analyses were performed to summarize the characteristics of bleeding and non-bleeding reports.

A two-by-two contingency table was constructed for each drug–event pair. reporting odds ratio (ROR) was used as the primary disproportionality measure, and the corresponding ROR values and 95% confidence intervals (CIs) were calculated. A positive ROR signal was defined as a PT with at least three reports (a ≥ 3), an ROR > 1, and a lower limit of the 95% CI exceeding 1 (13).

To further assess the consistency and robustness of the detected signals, three complementary disproportionality approaches, including the proportional reporting ratio (PRR), Bayesian confidence propagation neural network (BCPNN), and multi-item gamma Poisson shrinker (MGPS), were additionally applied. For PRR, a signal was defined as PRR ≥ 2, χ² ≥ 4, and a ≥ 3; for BCPNN, a signal was defined as IC025 > 0; and for MGPS, a signal was defined as EB05 > 2 and a > 0. The calculation formulas and signal detection criteria for the four algorithms are provided in **Supplementary Table 2**. Higher ROR values indicate stronger disproportional reporting of a specific adverse event for a given drug compared with other drugs in the FAERS database. The results of all four disproportionality algorithms are presented in **Supplementary Table 3**.

Categorical variables were summarized as frequencies and percentages and compared using the chi-square test. Continuous variables were summarized using medians and interquartile ranges (IQRs) where appropriate. All statistical analyses were conducted using R software (version 4.2.3), and a two-sided P value < 0.05 was considered statistically significant.

## 3 Results

### 3.1 Screening of bleeding adverse events

**Figure 1:**
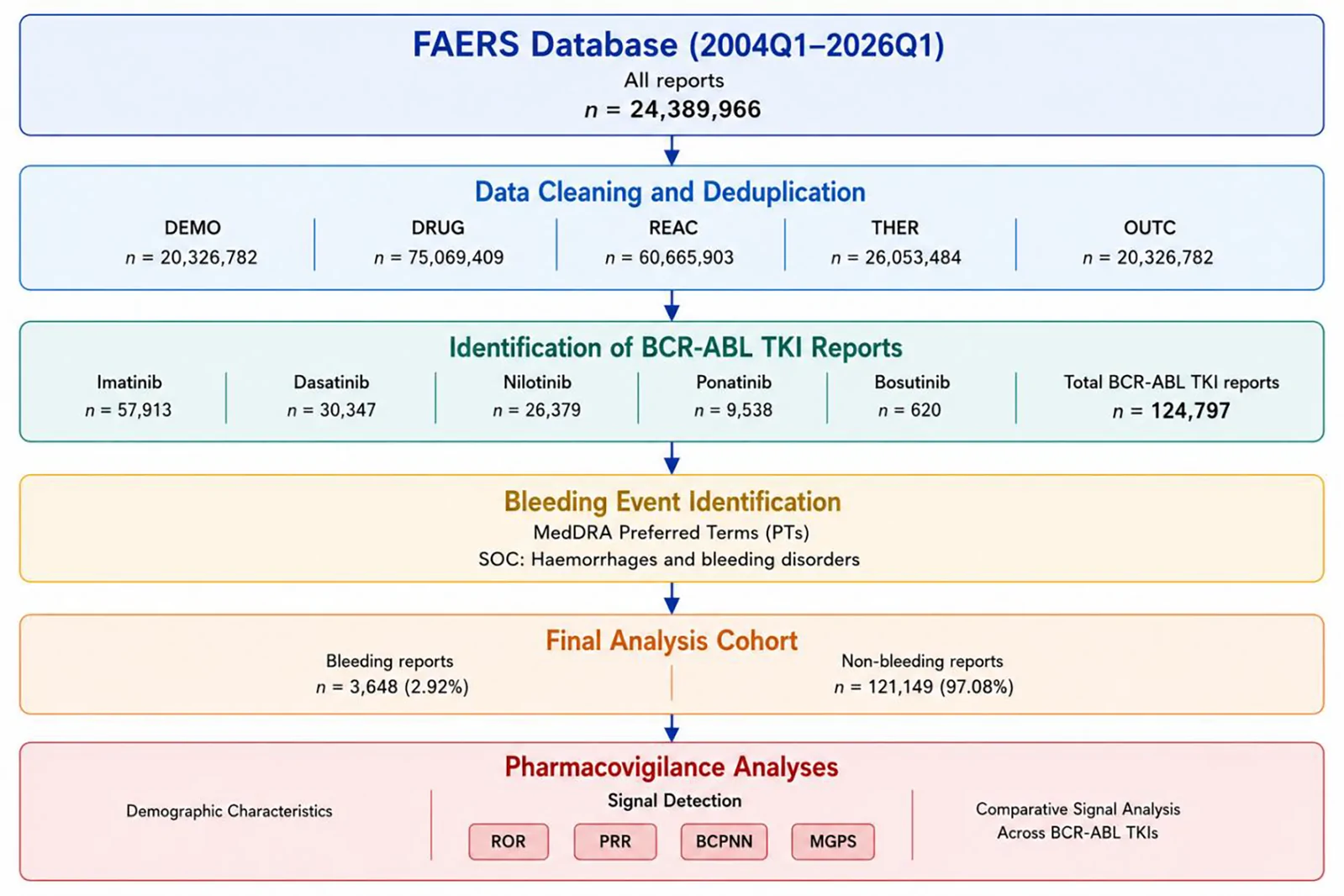
Flowchart of case selection and identification of bleeding events associated with BCR-ABL TKIs.

A total of 24, 389, 966 FAERS reports from 2004Q1 to 2026Q1 were retrieved, of which 20, 326, 782 unique reports remained after deduplication. Among these, 124, 797 reports involved the five BCR-ABL tyrosine kinase inhibitors, including imatinib, dasatinib, nilotinib, ponatinib, and bosutinib. Based on the predefined bleeding-related MedDRA PT list, 3, 648 bleeding reports and 121, 149 non-bleeding reports were identified. The case selection process is illustrated in **Figure 1**.

### 3.2 Demographic and clinical characteristics

The demographic and clinical characteristics of bleeding and non-bleeding reports are summarized in **Table 1**. Significant differences were observed in sex, age, body weight, reporter type, and serious clinical outcomes (all P < 0.001). Male patients accounted for 50.3% of bleeding reports compared with 47.8% of non-bleeding reports, while patients aged 65–85 years accounted for 24.5% and 19.2%, respectively.

**Table 1:** Demographic and clinical characteristics of bleeding cases associated with BCR-ABL TKIs in the FAERS database.

Characteristics	Bleeding (n=3, 648)	Non-bleeding (n=121, 149)	P value
Sex			<0.001
Female	1, 600 (43.9%)	53, 163 (43.9%)	
Male	1, 834 (50.3%)	57, 958 (47.8%)	
Missing	214 (5.9%)	10, 028 (8.3%)	
Age			<0.001
<18 years	78 (2.1%)	1, 879 (1.6%)	
18–64.9 years	1, 308 (35.9%)	39, 491 (32.6%)	
65–85 years	892 (24.5%)	23, 260 (19.2%)	
>85 years	40 (1.1%)	1, 668 (1.4%)	
Missing	1, 330 (36.5%)	54, 851 (45.3%)	
Weight			<0.001
<50 kg	97 (2.7%)	1474 (1.2%)	
50–100 kg	646 (17.7%)	12, 076 (10.0%)	
>100 kg	66 (1.8%)	1670 (1.4%)	
Missing	2, 839 (77.8%)	105, 929 (87.4%)	
Reporter type			<0.001
Consumer	1, 381 (37.9%)	48, 801 (40.3%)	
Health Professional	242 (6.6%)	10, 879 (9.0%)	
Lawyer	11 (0.3%)	303 (0.3%)	
Physician	1, 060 (29.1%)	31, 436 (25.9%)	
Other	510 (14.0%)	13, 972 (11.5%)	
Pharmacist	203 (5.6%)	8, 026 (6.6%)	
Registered Nurse	6 (0.2%)	143 (0.1%)	
Missing	235 (6.4%)	7, 589 (6.3%)	
Serious outcome			<0.001
Death	814 (22.3%)	26, 920 (22.2%)	
Hospitalization	724 (19.8%)	17, 411 (14.4%)	
Life-threatening	100 (2.7%)	1, 804 (1.5%)	
Other serious outcome	929 (25.5%)	28, 376 (23.4%)	
Disability	19 (0.5%)	620 (0.5%)	
Required intervention	2 (0.1%)	94 (0.1%)	
Congenital anomaly	0 (0.0%)	87 (0.1%)	
Missing	1, 060 (29.1%)	45, 837 (37.8%)	

Among clinical outcomes, hospitalization (19.8% vs. 14.4%) and life-threatening events (2.7% vs. 1.5%) were reported more frequently among bleeding than non-bleeding reports, whereas fatal outcomes were similar between the two groups (22.3% vs. 22.2%).

### 3.3 Distribution of bleeding events across SOCs

**Figure 2:**
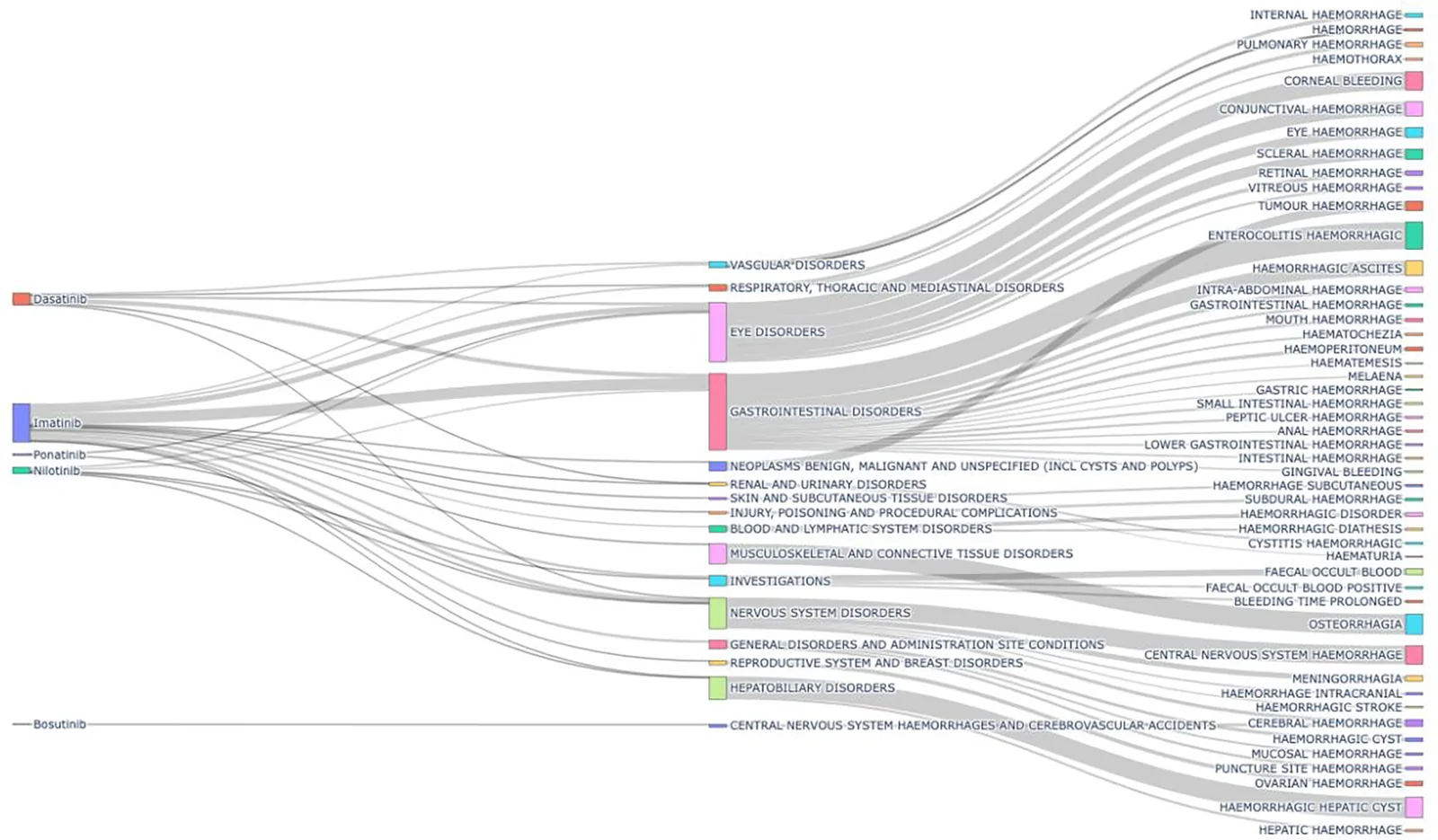
Sankey diagram illustrating the distribution of bleeding-related safety signals across BCR-ABL tyrosine kinase inhibitors (TKIs). The width of each flow is proportional to the number of reports associated with the corresponding pathway. The diagram highlights the contribution of different TKIs to organ-specific bleeding manifestations and their representative adverse event terms.

The distribution of bleeding-related adverse events across System Organ Classes (SOCs) and Preferred Terms (PTs) is presented in **Figure 2**. Bleeding events were reported across multiple organ systems, with gastrointestinal disorders, nervous system disorders, and eye disorders representing the most frequently involved SOCs.

Among gastrointestinal disorders, commonly reported PTs included gastrointestinal haemorrhage, haematochezia, haematemesis, melaena, and gastric haemorrhage. Nervous system disorders were mainly represented by cerebral haemorrhage and intracranial haemorrhage, whereas eye disorders included eye haemorrhage, conjunctival haemorrhage, and retinal haemorrhage.

Several SOCs contained relatively few PTs but demonstrated comparatively strong disproportionality signals, including hepatobiliary disorders, musculoskeletal and connective tissue disorders, and neoplasms benign, malignant and unspecified. Overall, bleeding manifestations were distributed across a broad range of organ systems, reflecting the heterogeneous nature of haemorrhagic adverse events associated with BCR-ABL TKIs.

### 3.4 Bleeding signal profiles of individual BCR-ABL TKIs

**Figure 3:**
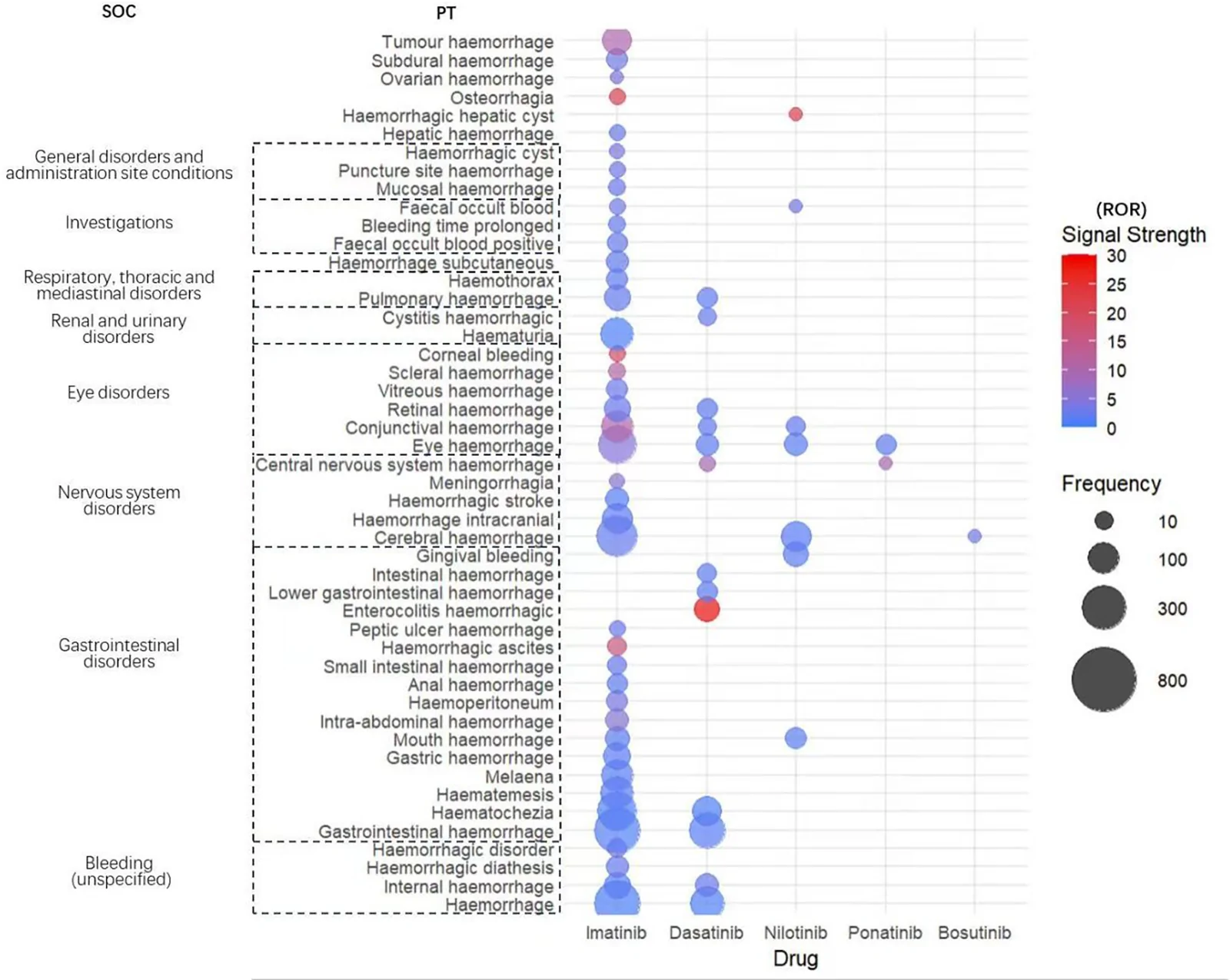
Distribution of positive bleeding signals across system organ classes (SOCs), preferred terms (PTs), and BCR-ABL tyrosine kinase inhibitors (TKIs). Bubble size represents the number of reports, and color intensity indicates signal strength measured by the reporting odds ratio (ROR). Only positive bleeding signals meeting the predefined signal detection criteria are displayed.

A total of 64 positive bleeding-related signals were identified across the five BCR-ABL TKIs. Imatinib accounted for the largest proportion of detected signals (41/64, 64.1%), followed by dasatinib (13/64, 20.3%), nilotinib (7/64, 10.9%), ponatinib (2/64, 3.1%), and bosutinib (1/64, 1.6%). Positive signals were detected for all five agents, although their distribution across SOCs and PTs varied substantially. Detailed signal profiles are presented in **Table 2** and **Figure 3**.

**Table 2:** Positive bleeding signals detected for BCR-ABL TKIs based on disproportionality algorithms.

SOC/Drug	Imatinib (n=41 signals)	Dasatinib (n=13 signals)	Nilotinib (n=7 signals)	Ponatinib (n=2 signals)	Bosutinib (n=1 signal)
Bleeding (unspecified)	Haemorrhage (334, ROR = 1.15) Internal haemorrhage (49, ROR = 1.54) Haemorrhagic diathesis (25, ROR = 2.50) Haemorrhagic disorder (13, ROR = 4.28)	Haemorrhage (138, ROR = 1.21) Internal haemorrhage (32, ROR = 2.56)	—	—	—
Gastrointestinal disorders	Gastrointestinal haemorrhage (329, ROR = 1.40) Haematochezia (202, ROR = 1.32) Haematemesis (119, ROR = 1.68) Melaena (109, ROR = 1.77) Gastric haemorrhage (57, ROR = 1.76) Mouth haemorrhage (36, ROR = 1.77) Intra-abdominal haemorrhage (34, ROR = 5.15) Haemoperitoneum (23, ROR = 3.80) Anal haemorrhage (15, ROR = 1.94) Small intestinal haemorrhage (14, ROR = 2.51) Haemorrhagic ascites (12, ROR = 15.77) Peptic ulcer haemorrhage (5, ROR = 2.62)	Gastrointestinal haemorrhage (160, ROR = 1.73) Haematochezia (78, ROR = 1.30) Enterocolitis haemorrhagic (46, ROR = 28.98) Lower gastrointestinal haemorrhage (16, ROR = 1.96) Intestinal haemorrhage (13, ROR = 2.10)	Gingival bleeding (44, ROR = 2.08) Mouth haemorrhage (21, ROR = 1.84)	—	—
Nervous system disorders	Cerebral haemorrhage (230, ROR = 2.32) Haemorrhage intracranial (84, ROR = 2.01) Haemorrhagic stroke (35, ROR = 1.59) Meningorrhagia (4, ROR = 5.78)	Central nervous system haemorrhage (5, ROR = 9.84)	Cerebral haemorrhage (89, ROR = 1.60)	Central nervous system haemorrhage (3, ROR = 9.82)	Cerebral haemorrhage (3, ROR = 3.33)
Eye disorders	Eye haemorrhage (185, ROR = 5.03) Conjunctival haemorrhage (110, ROR = 10.57) Retinal haemorrhage (51, ROR = 2.59) Vitreous haemorrhage (21, ROR = 2.72) Scleral haemorrhage (7, ROR = 11.07) Corneal bleeding (5, ROR = 19.42)	Eye haemorrhage (28, ROR = 1.92) Retinal haemorrhage (16, ROR = 2.06) Conjunctival haemorrhage (9, ROR = 2.14)	Eye haemorrhage (34, ROR = 1.64) Conjunctival haemorrhage (14, ROR = 2.35)	Eye haemorrhage (17, ROR = 1.94)	—
Renal and urinary disorders	Haematuria (122, ROR = 1.27)	Cystitis haemorrhagic (10, ROR = 2.28)	—	—	—
Respiratory, thoracic and mediastinal disorders	Pulmonary haemorrhage (49, ROR = 2.24) Haemothorax (20, ROR = 2.36)	Pulmonary haemorrhage (17, ROR = 1.98)	—	—	—
Skin and subcutaneous tissue disorders	Haemorrhage subcutaneous (24, ROR = 2.41)	—	—	—	—
Investigations	Faecal occult blood positive (16, ROR = 2.44) Bleeding time prolonged (8, ROR = 2.36) Faecal occult blood (5, ROR = 3.18)	—	Faecal occult blood (3, ROR = 3.42)	—	—
General disorders and administration site conditions	Mucosal haemorrhage (8, ROR = 2.48) Puncture site haemorrhage (6, ROR = 3.05) Haemorrhagic cyst (4, ROR = 3.72)	—	—	—	—
Hepatobiliary disorders	Hepatic haemorrhage (6, ROR = 2.50)	—	Haemorrhagic hepatic cyst (3, ROR = 21.89)	—	—
Musculoskeletal and connective tissue disorders	Osteorrhagia (5, ROR = 21.32)	—	—	—	—
Reproductive system and breast disorders	Ovarian haemorrhage (3, ROR = 4.66)	—	—	—	—
Injury, poisoning and procedural complications	Subdural haemorrhage (23, ROR = 3.00)	—	—	—	—
Neoplasms benign, malignant and unspecified	Tumour haemorrhage (76, ROR = 9.96)	—	—	—	—

Imatinib generated 41 positive signals across 13 SOCs (64.1% of all detected signals). Frequently reported signals included gastrointestinal haemorrhage (n = 329, ROR = 1.40), haematochezia (n = 202, ROR = 1.32), and cerebral haemorrhage (n = 230, ROR = 2.32). Several uncommon PTs showed high disproportionality estimates, including osteorrhagia (ROR = 21.32), corneal bleeding (ROR = 19.42), and tumour haemorrhage (ROR = 9.96), although these signals were based on relatively few reports.

Dasatinib generated 13 positive signals (20.3%), with gastrointestinal haemorrhage (n = 160, ROR = 1.73) among the most frequently reported events. Enterocolitis haemorrhagic showed a markedly elevated disproportionality estimate (n = 46, ROR = 28.98).

Nilotinib generated seven positive signals (10.9%) involving gastrointestinal, nervous system, eye, and hepatobiliary disorders. Cerebral haemorrhage was the most frequently reported neurological bleeding event (n = 89, ROR = 1.60), whereas haemorrhagic hepatic cyst showed a high ROR based on only three reports (ROR = 21.89).

Ponatinib and bosutinib accounted for two (3.1%) and one (1.6%) positive signals, respectively. For ponatinib, eye haemorrhage was reported in 17 cases (ROR = 1.94), while central nervous system haemorrhage was based on three reports (ROR = 9.82). Bosutinib was associated with cerebral haemorrhage (n = 3, ROR = 3.33).

Gastrointestinal haemorrhage, cerebral haemorrhage, and eye haemorrhage were recurrent PTs detected across multiple BCR-ABL TKIs. Overall, gastrointestinal disorders accounted for the largest proportion of detected signals, followed by nervous system and eye disorders. Rare signals with high disproportionality estimates should be interpreted cautiously because estimates based on small numbers of reports may be unstable. The distribution of positive signals across individual TKIs, SOCs, and PTs is shown in **Figure 3**.

The concordance of the four disproportionality algorithms is summarized in **Supplementary Table 3**. While some drug–event combinations were supported by all four algorithms, others were detected by fewer approaches, providing additional information on the robustness of individual signals.

## 4 Discussion

### 4.1 Overall bleeding profiles and signal characteristics

In this large-scale pharmacovigilance study based on the FAERS database from 2004Q1 to 2026Q1, we systematically characterized bleeding-related adverse event signals associated with five BCR-ABL tyrosine kinase inhibitors (TKIs), including imatinib, dasatinib, nilotinib, ponatinib, and bosutinib. A total of 64 positive bleeding signals were identified involving multiple organ systems. These findings provide an overview of the reported bleeding-related safety patterns associated with BCR-ABL TKI therapy.

Although the safety profiles of BCR-ABL TKIs have been extensively investigated, bleeding events have received relatively less attention than hematologic toxicities, cardiovascular complications, and treatment resistance (2, 7). In the present study, 3, 648 bleeding reports were identified among 124, 797 BCR-ABL TKI-related reports, representing 2.92% of the reports included in the analysis.

Hospitalization and life-threatening outcomes were reported more frequently among bleeding reports than among non-bleeding reports. However, these differences should be interpreted as descriptive reporting patterns rather than evidence that bleeding itself caused greater clinical severity. FAERS is subject to confounding, differential reporting, incomplete clinical information, and differences in the characteristics of individual reports. In particular, disease severity, concomitant medications, comorbidities, and other clinical factors may influence both the occurrence of bleeding and the reporting of serious outcomes. Previous clinical studies have reported severe haemorrhagic complications among patients receiving BCR-ABL TKIs, including treatment interruption, hospitalization, and other adverse clinical outcomes (7, 8). These findings support the importance of recognizing and appropriately managing bleeding events during TKI treatment, while the specific contribution of bleeding to clinical outcomes should be further evaluated in controlled clinical and epidemiological studies.

At the organ-system level, gastrointestinal disorders, nervous system disorders, and eye disorders represented the most frequently involved SOCs. Gastrointestinal haemorrhage, cerebral haemorrhage, and eye haemorrhage were recurrent PTs detected across multiple BCR-ABL TKIs. These findings indicate that haemorrhagic manifestations reported in association with BCR-ABL TKI therapy may involve a broad range of anatomical sites and clinical presentations. The observed distribution of signals across multiple SOCs further highlights the heterogeneous nature of bleeding-related adverse events reported in routine clinical practice.

### 4.2 Comparison with previous pharmacovigilance and clinical evidence

Our findings are broadly consistent with previous pharmacovigilance and clinical evidence, particularly for gastrointestinal bleeding. A study using the Japanese Adverse Drug Event Report (JADER) database identified significant gastrointestinal bleeding signals for dasatinib and imatinib, with adjusted RORs of 8.02 and 1.81, respectively (14). Clinical evidence also supports a clinically relevant bleeding phenotype associated with dasatinib. In a cohort of 138 patients with CML receiving dasatinib, bleeding occurred in 23% of patients, with gastrointestinal bleeding accounting for 81% of episodes; thrombocytopenia and advanced-phase disease were identified as important risk factors (8). In addition, impaired platelet aggregation has been demonstrated during dasatinib treatment, suggesting that platelet dysfunction may contribute to bleeding independently of thrombocytopenia (15). These findings provide clinical and mechanistic support for the gastrointestinal bleeding signals, particularly those associated with dasatinib, identified in the present FAERS analysis. However, differences in database characteristics, reporting behavior, patient populations, and exposure patterns preclude direct comparison of absolute bleeding risks across studies.

Previous FAERS-based studies have also characterized the broader safety profiles of imatinib, dasatinib, nilotinib, bosutinib, and ponatinib using disproportionality methods such as ROR and PRR, demonstrating distinct drug-specific safety patterns across BCR-ABL1 TKIs (16). Complementary evidence from VigiBase has similarly identified drug-specific safety signals among these agents, supporting the value of international spontaneous-reporting databases for post-marketing surveillance (17). Nevertheless, spontaneous-reporting analyses are hypothesis-generating and should be interpreted alongside clinical evidence rather than as estimates of comparative incidence or causality.

More recent evidence further highlights the complementary value of pharmacovigilance and real-world clinical evidence in evaluating treatment-related safety. A recent study integrating FAERS and the Canada Vigilance Database demonstrated the value of multiple spontaneous-reporting databases for identifying and cross-validating potential adverse-event signals (18). Complementary real-world clinical studies have also emphasized the importance of longitudinal clinical characteristics and patient outcomes when interpreting treatment-related safety findings (19–21). Although these studies were conducted in different disease and treatment settings, they support an integrated interpretation of spontaneous-reporting signals alongside real-world clinical evidence.

The mechanisms underlying bleeding complications associated with BCR-ABL TKIs are likely multifactorial. Previous studies have demonstrated that BCR-ABL TKIs may impair platelet function through inhibition of signaling pathways involved in platelet activation and aggregation (8, 22). Experimental evidence has shown that both imatinib and dasatinib can induce reversible platelet dysfunction, particularly through impairment of arachidonic acid- and epinephrine-mediated platelet aggregation (15). In addition, thrombocytopenia remains one of the most common hematologic toxicities associated with TKI therapy and may directly increase bleeding susceptibility (23).

Among the identified signals, gastrointestinal haemorrhage represented one of the most frequently reported bleeding manifestations. Several studies have reported clinically significant gastrointestinal bleeding during TKI treatment, particularly in patients receiving dasatinib (8). Dasatinib inhibits multiple Src-family kinases in addition to BCR-ABL, and Src-family kinases play important roles in platelet activation, endothelial integrity, and vascular permeability (8, 24). Consequently, dasatinib-related bleeding may involve mechanisms extending beyond thrombocytopenia alone. Previous clinical studies have similarly reported increased bleeding tendency and platelet dysfunction in patients receiving dasatinib, even in the absence of severe thrombocytopenia (16, 18).

Neurological bleeding events, including cerebral haemorrhage and intracranial haemorrhage, were also detected. Although these events were reported less frequently than gastrointestinal bleeding, they may be associated with considerable clinical consequences because of their potentially life-threatening nature. Similarly, ocular bleeding events were observed across multiple agents, indicating that haemorrhagic manifestations may occur in diverse anatomical locations.

Several uncommon PTs demonstrated elevated disproportionality estimates. However, many of these signals were based on relatively small numbers of reports and should therefore be interpreted cautiously. Signal amplification may occur when rare adverse events are reported only a few times, potentially resulting in unstable disproportionality estimates (25). Additional observational and mechanistic studies are required to determine whether these rare signals represent true drug-related adverse reactions or reflect reporting variability.

### 4.3 Clinical implications

The present findings have several important clinical implications. First, bleeding events should be recognized as a potentially important component of the overall safety profile of BCR-ABL TKIs. Although severe bleeding appears relatively uncommon, clinically significant haemorrhagic complications may occur and may result in hospitalization or life-threatening outcomes (7, 8).

Second, clinicians should remain vigilant when prescribing BCR-ABL TKIs to patients with pre-existing bleeding disorders, thrombocytopenia, gastrointestinal disease, cerebrovascular disease, or concomitant anticoagulant and antiplatelet therapy (26). Routine monitoring of platelet counts and careful assessment of bleeding symptoms may facilitate earlier detection and intervention.

Finally, pharmacovigilance analyses provide complementary information to clinical trials by identifying rare, delayed, and heterogeneous adverse events that may not be fully captured during pre-marketing development (10, 27). The present study expands current knowledge regarding bleeding-related adverse events associated with BCR-ABL TKIs and may provide useful information for future epidemiological and mechanistic investigations.

Beyond conventional molecular response assessment, emerging complementary biomarkers may further support personalized monitoring of patients with CML receiving BCR-ABL TKIs. A recent study reported an inverse relationship between extracellular vesicle-associated BCR::ABL1 transcripts and circulating CD26+ leukemic stem cells, particularly among patients achieving deep molecular responses, suggesting that these biomarkers may provide complementary information on residual disease dynamics (28). Although this emerging approach is not directly related to bleeding toxicity, integrating disease-monitoring biomarkers with pharmacovigilance information may ultimately contribute to more individualized management of patients receiving BCR-ABL TKIs. Further studies are required to establish the clinical utility and prognostic value of these biomarkers.

### 4.4 Limitations

Several limitations inherent to FAERS-based analyses should be acknowledged. First, FAERS is a spontaneous reporting system and is therefore subject to underreporting, reporting bias, duplicate reporting, and incomplete clinical information (10, 27). Consequently, causal relationships between drug exposure and adverse events cannot be established.

Second, disproportionality analyses identify statistical associations rather than incidence rates or absolute risks (10, 25, 27). Because FAERS lacks denominator information regarding the total number of exposed patients, direct comparisons of bleeding risk among different TKIs cannot be performed. Furthermore, differences in reporting frequency may be influenced by drug utilization, market availability, reporting practices, and channeling bias.

Third, potential confounding by indication and concomitant medications could not be adequately controlled in the present analysis. Anticoagulants and antiplatelet agents may independently increase the likelihood of bleeding, while thrombocytopenia and other disease- or treatment-related hematological abnormalities may also contribute to haemorrhagic events. In addition, disease severity, treatment line, prior therapies, treatment duration, and treatment indications may influence both TKI selection and the occurrence or reporting of bleeding events. However, FAERS does not consistently provide sufficiently detailed information on these clinical factors, including disease status, treatment line, laboratory findings, and complete concomitant medication histories. Therefore, residual confounding cannot be excluded, and the observed disproportionality signals should not be interpreted as effects attributable solely to the BCR-ABL TKIs.

Despite these limitations, the large sample size, long observation period, and comprehensive evaluation of bleeding-related adverse event signals provide valuable real-world insights into the haemorrhagic safety profiles associated with BCR-ABL TKIs.

## 5 Conclusions

Using real-world data from the FAERS database, this study comprehensively characterized bleeding-related adverse event signals associated with five BCR-ABL tyrosine kinase inhibitors. A total of 64 positive bleeding signals were identified, involving diverse organ systems and clinical manifestations. Gastrointestinal, nervous system, and ocular haemorrhagic events accounted for the majority of detected signals.

Although causality and comparative risk cannot be established using spontaneous reporting data, the present findings expand current knowledge regarding the haemorrhagic safety profiles of BCR-ABL TKIs and provide valuable evidence for pharmacovigilance practice. Continued monitoring and further clinical investigations are needed to better understand the mechanisms, risk factors, and clinical impact of bleeding-related adverse events during BCR-ABL TKI therapy.

## Data Availability

Publicly available datasets were analyzed in this study. This data can be found here: This study is based on the FAERS database which is publicly accessible at https://www.fda.gov/drugs/surveillance/fdas-adverse-event-reporting-system-faers. Further information is available from the corresponding author upon request.
