# Supplementary material for: Characterizing bleeding adverse events associated with BCR-ABL tyrosine kinase inhibitors: insights from FAERS reports
Source: Front Oncol. 2026 Sep 9;16:1912458. doi: 10.3389/fonc.2026.1912458 (PMC13597428; doi:10.3389/fonc.2026.1912458)
Supplement: Supplementary file 1 [file Table1.docx]

**Supplementary Table S1. The final list of bleeding-related PTs**

| **System Organ Class (SOC)** | **Preferred Term (PT)** |
| --- | --- |
| VASCULAR DISORDERS | HAEMORRHAGE HAEMATOMA INTERNAL HAEMORRHAGE SHOCK HAEMORRHAGIC ARTERIAL HAEMORRHAGE BLOODY DISCHARGE VENOUS HAEMORRHAGE |
| GASTROINTESTINAL DISORDERS | GASTROINTESTINAL HAEMORRHAGE HAEMATOCHEZIA HAEMATEMESIS RECTAL HAEMORRHAGE GASTRIC HAEMORRHAGE GINGIVAL BLEEDING UPPER GASTROINTESTINAL HAEMORRHAGE MOUTH HAEMORRHAGE INTRA-ABDOMINAL HAEMORRHAGE HAEMOPERITONEUM DIARRHOEA HAEMORRHAGIC HAEMORRHOIDAL HAEMORRHAGE INTESTINAL HAEMORRHAGE ANAL HAEMORRHAGE SMALL INTESTINAL HAEMORRHAGE GASTRIC ULCER HAEMORRHAGE HAEMORRHAGIC ASCITES LOWER GASTROINTESTINAL HAEMORRHAGE LARGE INTESTINAL HAEMORRHAGE RETROPERITONEAL HAEMORRHAGE PEPTIC ULCER HAEMORRHAGE TONGUE HAEMORRHAGE GASTRITIS HAEMORRHAGIC LIP HAEMORRHAGE OESOPHAGEAL HAEMORRHAGE OESOPHAGEAL VARICES HAEMORRHAGE RETROPERITONEAL HAEMATOMA ABDOMINAL WALL HAEMORRHAGE DIVERTICULUM INTESTINAL HAEMORRHAGIC TOOTH SOCKET HAEMORRHAGE MESENTERIC HAEMATOMA PERITONEAL HAEMATOMA HAEMORRHAGIC EROSIVE GASTRITIS GASTRODUODENAL HAEMORRHAGE OESOPHAGEAL ULCER HAEMORRHAGE PANCREATITIS HAEMORRHAGIC MELAENA PROCTITIS HAEMORRHAGIC ENTEROCOLITIS HAEMORRHAGIC GASTROINTESTINAL ULCER HAEMORRHAGE LARGE INTESTINAL ULCER HAEMORRHAGE DUODENAL ULCER HAEMORRHAGE |
| NERVOUS SYSTEM DISORDERS | CEREBRAL HAEMORRHAGE HAEMORRHAGE INTRACRANIAL HAEMORRHAGIC STROKE SUBARACHNOID HAEMORRHAGE CEREBRAL HAEMATOMA MENINGORRHAGIA INTRACRANIAL HAEMATOMA BRAIN STEM HAEMORRHAGE CENTRAL NERVOUS SYSTEM HAEMORRHAGE INTRAVENTRICULAR HAEMORRHAGE SPINAL CORD HAEMATOMA CEREBELLAR HAEMORRHAGE BRAIN STEM HAEMATOMA THALAMUS HAEMORRHAGE SPINAL CORD HAEMORRHAGE BASAL GANGLIA HAEMORRHAGE SPINAL EPIDURAL HAEMORRHAGE PUTAMEN HAEMORRHAGE ACUTE HAEMORRHAGIC LEUKOENCEPHALITIS CEREBRAL MICROHAEMORRHAGE |
| EYE DISORDERS | EYE HAEMORRHAGE CONJUNCTIVAL HAEMORRHAGE RETINAL HAEMORRHAGE VITREOUS HAEMORRHAGE SCLERAL HAEMORRHAGE CORNEAL BLEEDING RETINAL ISCHAEMIA EYELID BLEEDING IRIS HAEMORRHAGE LACRIMAL HAEMORRHAGE EYE HAEMATOMA SPONTANEOUS HYPHAEMA SUBRETINAL HAEMATOMA RETINOPATHY HAEMORRHAGIC |
| RENAL AND URINARY DISORDERS | HAEMATURIA CYSTITIS HAEMORRHAGIC URINARY BLADDER HAEMORRHAGE RENAL HAEMORRHAGE HAEMORRHAGE URINARY TRACT URETHRAL HAEMORRHAGE RENAL CYST HAEMORRHAGE URETERIC HAEMORRHAGE UROGENITAL HAEMORRHAGE |
| RESPIRATORY, THORACIC AND MEDIASTINAL DISORDERS | EPISTAXIS PULMONARY CONGESTION PULMONARY HAEMORRHAGE HAEMOTHORAX PULMONARY ALVEOLAR HAEMORRHAGE LARYNGEAL HAEMORRHAGE PHARYNGEAL HAEMORRHAGE RESPIRATORY TRACT HAEMORRHAGE BRONCHIAL HAEMORRHAGE HAEMOPTYSIS THORACIC HAEMORRHAGE |
| NEOPLASMS BENIGN, MALIGNANT AND UNSPECIFIED (INCL CYSTS AND POLYPS) | TUMOUR HAEMORRHAGE |
| REPRODUCTIVE SYSTEM AND BREAST DISORDERS | VAGINAL HAEMORRHAGE HEAVY MENSTRUAL BLEEDING INTERMENSTRUAL BLEEDING GENITAL HAEMORRHAGE UTERINE HAEMORRHAGE OVARIAN HAEMORRHAGE TESTICULAR HAEMORRHAGE POSTMENOPAUSAL HAEMORRHAGE PELVIC HAEMORRHAGE BREAST HAEMORRHAGE CERVIX HAEMORRHAGE UTERINE ABNORMAL UTERINE BLEEDING |
| INVESTIGATIONS | BLOOD URINE PRESENT FAECAL OCCULT BLOOD POSITIVE BLEEDING TIME PROLONGED FAECAL OCCULT BLOOD |
| SKIN AND SUBCUTANEOUS TISSUE DISORDERS | SKIN HAEMORRHAGE HAEMORRHAGE SUBCUTANEOUS SKIN ULCER HAEMORRHAGE NAIL BED BLEEDING HAEMORRHAGE SUBEPIDERMAL MUCOCUTANEOUS HAEMORRHAGE SPLINTER HAEMORRHAGES |
| BLOOD AND LYMPHATIC SYSTEM DISORDERS | HAEMORRHAGIC DIATHESIS HAEMORRHAGIC DISORDER SPLENIC HAEMORRHAGE SPONTANEOUS HAEMORRHAGE |
| GENERAL DISORDERS AND ADMINISTRATION SITE CONDITIONS | ULCER HAEMORRHAGE MUCOSAL HAEMORRHAGE PUNCTURE SITE HAEMORRHAGE HAEMORRHAGIC CYST CATHETER SITE HAEMORRHAGE VESSEL PUNCTURE SITE HAEMORRHAGE INJECTION SITE HAEMORRHAGE |
| INJURY, POISONING AND PROCEDURAL COMPLICATIONS | SUBDURAL HAEMORRHAGE POST PROCEDURAL HAEMORRHAGE WOUND HAEMORRHAGE INCISION SITE HAEMORRHAGE EPIDURAL HAEMORRHAGE PERIORBITAL HAEMORRHAGE TRACHEAL HAEMORRHAGE ANASTOMOTIC HAEMORRHAGE TRAUMATIC HAEMORRHAGE STOMA SITE HAEMORRHAGE TRAUMATIC INTRACRANIAL HAEMORRHAGE VASCULAR ACCESS SITE HAEMORRHAGE |
| MUSCULOSKELETAL AND CONNECTIVE TISSUE DISORDERS | HAEMARTHROSIS MUSCLE HAEMORRHAGE OSTEORRHAGIA SOFT TISSUE HAEMORRHAGE |
| PREGNANCY, PUERPERIUM AND PERINATAL CONDITIONS | RETROPLACENTAL HAEMATOMA POSTPARTUM HAEMORRHAGE HAEMORRHAGE IN PREGNANCY PERIPARTUM HAEMORRHAGE INTRAPARTUM HAEMORRHAGE POST ABORTION HAEMORRHAGE |
| HEPATOBILIARY DISORDERS | HEPATIC HAEMORRHAGE HEPATIC HAEMATOMA HAEMOBILIA HAEMORRHAGIC HEPATIC CYST |
| CARDIAC DISORDERS | PERICARDIAL HAEMORRHAGE PAPILLARY MUSCLE HAEMORRHAGE |
| EAR AND LABYRINTH DISORDERS | EAR HAEMORRHAGE |
| INFECTIONS AND INFESTATIONS | DENGUE HAEMORRHAGIC FEVER ADENOVIRAL HAEMORRHAGIC CYSTITIS |
| ENDOCRINE DISORDERS | ADRENAL HAEMORRHAGE PITUITARY HAEMORRHAGE |

**Supplementary Table S2. Four major algorithms used for signal detection.**

| **Algorithms** | **Equation** | **Criteria** |
| --- | --- | --- |
| ROR | ROR=ad/b/c | lower limit of 95% CI>1, a≥3 |
|  | 95%CI=e^ln(ROR)±1.96(1/a+1/b+1/c+1/d)^0.5^ |  |
| PRR | PRR=a(c+d)/c/(a+b) | PRR≥2, χ^2^≥4, a≥3 |
|  | χ^2^=[(ad-bc)^2](a+b+c+d)/[(a+b)(c+d)(a+c)(b+d)] |  |
| BCPNN | IC=log_2_a(a+b+c+d)(a+c)(a+b) | IC025>0 |
|  | 95%CI= E(IC) ± 2V(IC)^0.5 |  |
| MGPS | EBGM=a(a+b+c+d)/(a+c)/(a+b) | EBGM05>2 |
|  | 95%CI=e^ln(EBGM)±1.96(1/a+1/b+1/c+1/d)^0.5^ |  |

Caption: a, number of reports containing both the target drug and target adverse drug reaction; b, number of reports containing other adverse drug reaction of the target drug; c, number of reports containing the target adverse drug reaction of other drugs; d, number of reports containing other drugs and other adverse drug reactions. 95%CI, 95% confidence interval; χ2, chi-squared; IC, information component; IC025, the lower limit of 95% CI of the IC; E(IC), the IC expectations; V(IC), the variance of IC; EBGM, empirical Bayesian geometric mean; EBGM05, the lower limit of 95% CI of EBGM.

**Supplementary Table S3. Concordance of Four Disproportionality Algorithms for Bleeding-Related Drug–Event Combinations**

| Drug | PT | a | SOC | ROR (95% CI) | PRR (χ²) | EBGM (EB05) | IC (IC025) | Positive algorithms |
| --- | --- | --- | --- | --- | --- | --- | --- | --- |
| Imatinib | Haemorrhage | 334 | Vascular disorders | 1.15 (1.03-1.28)* | 1.15 (6.65) | 1.15 (1.03) | 0.2 (0.04)* | 2/4 |
| Imatinib | Gastrointestinal haemorrhage | 329 | Gastrointestinal disorders | 1.4 (1.26-1.56)* | 1.4 (37.32) | 1.4 (1.25) | 0.48 (0.32)* | 2/4 |
| Imatinib | Cerebral haemorrhage | 230 | Nervous system disorders | 2.32 (2.04-2.64)* | 2.32 (170.95)* | 2.31 (2.03)* | 1.21 (1.01)* | 4/4 |
| Imatinib | Haematochezia | 202 | Gastrointestinal disorders | 1.32 (1.15-1.52)* | 1.32 (16) | 1.32 (1.15) | 0.4 (0.2)* | 2/4 |
| Imatinib | Eye haemorrhage | 185 | Eye disorders | 5.03 (4.35-5.82)* | 5.03 (588.44)* | 4.97 (4.3)* | 2.31 (2.07)* | 4/4 |
| Imatinib | Haematuria | 122 | Renal and urinary disorders | 1.27 (1.07-1.52)* | 1.27 (7.1) | 1.27 (1.06) | 0.35 (0.08)* | 2/4 |
| Imatinib | Haematemesis | 119 | Gastrointestinal disorders | 1.68 (1.4-2.01)* | 1.68 (32.7) | 1.68 (1.4) | 0.75 (0.47)* | 2/4 |
| Imatinib | Conjunctival haemorrhage | 110 | Eye disorders | 10.57 (8.74-12.78)* | 10.56 (924.57)* | 10.28 (8.51)* | 3.36 (2.97)* | 4/4 |
| Imatinib | Melaena | 109 | Gastrointestinal disorders | 1.77 (1.47-2.14)* | 1.77 (36.4) | 1.77 (1.46) | 0.82 (0.54)* | 2/4 |
| Imatinib | Haemorrhage intracranial | 84 | Nervous system disorders | 2.01 (1.62-2.49)* | 2.01 (42.23)* | 2 (1.62) | 1 (0.67)* | 3/4 |
| Imatinib | Tumour haemorrhage | 76 | Neoplasms benign, malignant and unspecified (incl cysts and polyps) | 9.96 (7.93-12.51)* | 9.95 (595.22)* | 9.71 (7.73)* | 3.28 (2.79)* | 4/4 |
| Imatinib | Gastric haemorrhage | 57 | Gastrointestinal disorders | 1.76 (1.36-2.28)* | 1.76 (18.59) | 1.76 (1.35) | 0.81 (0.41)* | 2/4 |
| Imatinib | Retinal haemorrhage | 51 | Eye disorders | 2.59 (1.97-3.41)* | 2.59 (49.4)* | 2.58 (1.96) | 1.37 (0.92)* | 3/4 |
| Imatinib | Pulmonary haemorrhage | 49 | Respiratory, thoracic and mediastinal disorders | 2.24 (1.69-2.97)* | 2.24 (33.49)* | 2.23 (1.69) | 1.16 (0.71)* | 3/4 |
| Imatinib | Internal haemorrhage | 49 | Vascular disorders | 1.54 (1.16-2.04)* | 1.54 (9.3) | 1.54 (1.16) | 0.62 (0.2)* | 2/4 |
| Imatinib | Mouth haemorrhage | 36 | Gastrointestinal disorders | 1.77 (1.27-2.45)* | 1.77 (11.88) | 1.76 (1.27) | 0.82 (0.31)* | 2/4 |
| Imatinib | Haemorrhagic stroke | 35 | Nervous system disorders | 1.59 (1.14-2.22)* | 1.59 (7.71) | 1.59 (1.14) | 0.67 (0.16)* | 2/4 |
| Imatinib | Intra-abdominal haemorrhage | 34 | Gastrointestinal disorders | 5.15 (3.67-7.22)* | 5.15 (111.97)* | 5.09 (3.63)* | 2.35 (1.7)* | 4/4 |
| Imatinib | Haemorrhagic diathesis | 25 | Blood and lymphatic system disorders | 2.5 (1.69-3.71)* | 2.5 (22.41)* | 2.49 (1.68) | 1.32 (0.67)* | 3/4 |
| Imatinib | Haemorrhage subcutaneous | 24 | Skin and subcutaneous tissue disorders | 2.41 (1.62-3.61)* | 2.41 (19.74)* | 2.4 (1.61) | 1.27 (0.61)* | 3/4 |
| Imatinib | Haemoperitoneum | 23 | Gastrointestinal disorders | 3.8 (2.52-5.73)* | 3.8 (46.86)* | 3.77 (2.5)* | 1.91 (1.16)* | 4/4 |
| Imatinib | Subdural haemorrhage | 23 | Injury, poisoning and procedural complications | 3 (1.99-4.52)* | 3 (30.43)* | 2.98 (1.98) | 1.58 (0.87)* | 3/4 |
| Imatinib | Vitreous haemorrhage | 21 | Eye disorders | 2.72 (1.77-4.18)* | 2.72 (22.63)* | 2.7 (1.76) | 1.44 (0.71)* | 3/4 |
| Imatinib | Haemothorax | 20 | Respiratory, thoracic and mediastinal disorders | 2.36 (1.52-3.67)* | 2.36 (15.6)* | 2.35 (1.52) | 1.23 (0.51)* | 3/4 |
| Imatinib | Faecal occult blood positive | 16 | Investigations | 2.44 (1.49-3.99)* | 2.44 (13.51)* | 2.43 (1.49) | 1.28 (0.46)* | 3/4 |
| Imatinib | Anal haemorrhage | 15 | Gastrointestinal disorders | 1.94 (1.17-3.22)* | 1.94 (6.75) | 1.93 (1.16) | 0.95 (0.14)* | 2/4 |
| Imatinib | Small intestinal haemorrhage | 14 | Gastrointestinal disorders | 2.51 (1.48-4.24)* | 2.51 (12.59)* | 2.5 (1.48) | 1.32 (0.43)* | 3/4 |
| Imatinib | Haemorrhagic disorder | 13 | Blood and lymphatic system disorders | 4.28 (2.47-7.39)* | 4.28 (32.22)* | 4.24 (2.45)* | 2.08 (1.01)* | 4/4 |
| Imatinib | Haemorrhagic ascites | 12 | Gastrointestinal disorders | 15.77 (8.84-28.12)* | 15.77 (158.84)* | 15.13 (8.49)* | 3.92 (2.04)* | 4/4 |
| Imatinib | Mucosal haemorrhage | 8 | General disorders and administration site conditions | 2.48 (1.24-4.98)* | 2.48 (7.04)* | 2.47 (1.23) | 1.31 (0.12)* | 3/4 |
| Imatinib | Bleeding time prolonged | 8 | Investigations | 2.36 (1.18-4.73)* | 2.36 (6.24)* | 2.35 (1.17) | 1.23 (0.07)* | 3/4 |
| Imatinib | Scleral haemorrhage | 7 | Eye disorders | 11.07 (5.22-23.5)* | 11.07 (62.17)* | 10.76 (5.07)* | 3.43 (1.24)* | 4/4 |
| Imatinib | Puncture site haemorrhage | 6 | General disorders and administration site conditions | 3.05 (1.36-6.81)* | 3.05 (8.19)* | 3.03 (1.36) | 1.6 (0.13)* | 3/4 |
| Imatinib | Hepatic haemorrhage | 6 | Hepatobiliary disorders | 2.5 (1.12-5.59)* | 2.5 (5.38)* | 2.49 (1.12) | 1.32 (-0.06) | 2/4 |
| Imatinib | Osteorrhagia | 5 | Musculoskeletal and connective tissue disorders | 21.32 (8.64-52.58)* | 21.32 (91.25)* | 20.15 (8.17)* | 4.33 (1.04)* | 4/4 |
| Imatinib | Corneal bleeding | 5 | Eye disorders | 19.42 (7.89-47.8)* | 19.42 (82.76)* | 18.45 (7.5)* | 4.21 (1.02)* | 4/4 |
| Imatinib | Faecal occult blood | 5 | Investigations | 3.18 (1.32-7.68)* | 3.18 (7.42)* | 3.16 (1.31) | 1.66 (0.03)* | 3/4 |
| Imatinib | Peptic ulcer haemorrhage | 5 | Gastrointestinal disorders | 2.62 (1.09-6.33)* | 2.62 (4.99)* | 2.61 (1.08) | 1.39 (-0.14) | 2/4 |
| Imatinib | Meningorrhagia | 4 | Nervous system disorders | 5.78 (2.15-15.52)* | 5.78 (15.55)* | 5.7 (2.12)* | 2.51 (0.25)* | 4/4 |
| Imatinib | Haemorrhagic cyst | 4 | General disorders and administration site conditions | 3.72 (1.39-9.96)* | 3.72 (7.87)* | 3.69 (1.38) | 1.88 (-0.04) | 2/4 |
| Imatinib | Ovarian haemorrhage | 3 | Reproductive system and breast disorders | 4.66 (1.49-14.56)* | 4.66 (8.51)* | 4.61 (1.48) | 2.21 (-0.18) | 2/4 |
| Dasatinib | Gastrointestinal haemorrhage | 160 | Gastrointestinal disorders | 1.73 (1.48-2.02)* | 1.73 (49.38) | 1.73 (1.48) | 0.79 (0.56)* | 2/4 |
| Dasatinib | Haemorrhage | 138 | Vascular disorders | 1.21 (1.02-1.43)* | 1.21 (5.07) | 1.21 (1.02) | 0.28 (0.03)* | 2/4 |
| Dasatinib | Haematochezia | 78 | Gastrointestinal disorders | 1.3 (1.04-1.63)* | 1.3 (5.43) | 1.3 (1.04) | 0.38 (0.05)* | 2/4 |
| Dasatinib | Enterocolitis haemorrhagic | 46 | Gastrointestinal disorders | 28.98 (21.6-38.87)* | 28.96 (1202.55)* | 28.08 (20.93)* | 4.81 (3.73)* | 4/4 |
| Dasatinib | Internal haemorrhage | 32 | Vascular disorders | 2.56 (1.81-3.63)* | 2.56 (30.44)* | 2.56 (1.81) | 1.36 (0.79)* | 3/4 |
| Dasatinib | Eye haemorrhage | 28 | Eye disorders | 1.92 (1.32-2.78)* | 1.92 (12.25) | 1.91 (1.32) | 0.94 (0.36)* | 2/4 |
| Dasatinib | Pulmonary haemorrhage | 17 | Respiratory, thoracic and mediastinal disorders | 1.98 (1.23-3.18)* | 1.97 (8.16) | 1.97 (1.23) | 0.98 (0.22)* | 2/4 |
| Dasatinib | Retinal haemorrhage | 16 | Eye disorders | 2.06 (1.26-3.37)* | 2.06 (8.72)* | 2.06 (1.26) | 1.04 (0.25)* | 3/4 |
| Dasatinib | Lower gastrointestinal haemorrhage | 16 | Gastrointestinal disorders | 1.96 (1.2-3.21)* | 1.96 (7.53) | 1.96 (1.2) | 0.97 (0.19)* | 2/4 |
| Dasatinib | Intestinal haemorrhage | 13 | Gastrointestinal disorders | 2.1 (1.22-3.63)* | 2.1 (7.51)* | 2.1 (1.22) | 1.07 (0.19)* | 3/4 |
| Dasatinib | Cystitis haemorrhagic | 10 | Renal and urinary disorders | 2.28 (1.22-4.23)* | 2.28 (7.13)* | 2.27 (1.22) | 1.18 (0.15)* | 3/4 |
| Dasatinib | Conjunctival haemorrhage | 9 | Eye disorders | 2.14 (1.11-4.13)* | 2.14 (5.48)* | 2.14 (1.11) | 1.1 (0.03)* | 3/4 |
| Dasatinib | Central nervous system haemorrhage | 5 | Nervous system disorders | 9.84 (4.08-23.75)* | 9.84 (39.27)* | 9.74 (4.04)* | 3.28 (0.8)* | 4/4 |
| Nilotinib | Cerebral haemorrhage | 89 | Nervous system disorders | 1.6 (1.3-1.97)* | 1.6 (20.09) | 1.6 (1.3) | 0.68 (0.36)* | 2/4 |
| Nilotinib | Gingival bleeding | 44 | Gastrointestinal disorders | 2.08 (1.54-2.79)* | 2.08 (24.46)* | 2.07 (1.54) | 1.05 (0.59)* | 3/4 |
| Nilotinib | Eye haemorrhage | 34 | Eye disorders | 1.64 (1.17-2.3)* | 1.64 (8.45) | 1.64 (1.17) | 0.71 (0.2)* | 2/4 |
| Nilotinib | Mouth haemorrhage | 21 | Gastrointestinal disorders | 1.84 (1.2-2.83)* | 1.84 (8.09) | 1.84 (1.2) | 0.88 (0.21)* | 2/4 |
| Nilotinib | Conjunctival haemorrhage | 14 | Eye disorders | 2.35 (1.39-3.97)* | 2.35 (10.83)* | 2.35 (1.39) | 1.23 (0.36)* | 3/4 |
| Nilotinib | Faecal occult blood | 3 | Investigations | 3.42 (1.1-10.62)* | 3.42 (5.1)* | 3.4 (1.09) | 1.77 (-0.36) | 2/4 |
| Nilotinib | Haemorrhagic hepatic cyst | 3 | Hepatobiliary disorders | 21.89 (6.92-69.2)* | 21.89 (57.78)* | 21.18 (6.7)* | 4.4 (0.33) | 3/4 |
| Ponatinib | Eye haemorrhage | 17 | Eye disorders | 1.94 (1.21-3.13)* | 1.94 (7.76) | 1.94 (1.21) | 0.96 (0.2)* | 2/4 |
| Ponatinib | Central nervous system haemorrhage | 3 | Nervous system disorders | 9.82 (3.15-30.55)* | 9.82 (23.6)* | 9.76 (3.14)* | 3.29 (0.16)* | 4/4 |
| Bosutinib | Cerebral haemorrhage | 3 | Central nervous system haemorrhages and cerebrovascular accidents | 3.33 (1.07-10.32)* | 3.32 (4.87)* | 3.32 (1.07) | 1.73 (-0.37) | 2/4 |

*: The asterisk indicates that the corresponding disproportionality algorithm met its predefined signal detection criterion. ROR was used as the primary disproportionality measure; therefore, all drug–event combinations listed in this table met the ROR criterion. PRR, BCPNN (IC), and MGPS (EBGM) were used as complementary approaches. 'Positive algorithms' indicates the number of algorithms meeting their predefined criteria out of four.

**Note:** The table presents drug–event combinations meeting the predefined ROR signal criterion and/or identified as positive by at least one complementary algorithm. ROR was used as the primary disproportionality measure, while PRR, BCPNN, and MGPS were applied as complementary approaches. An asterisk (*) indicates that the corresponding algorithm met its predefined signal detection criterion. The 'Positive algorithms' column summarizes the number of positive algorithms among ROR, PRR, BCPNN, and MGPS.
